# Effects of nasal high flow on nocturnal hypercapnia, sleep, and sympathovagal balance in patients with neuromuscular disorders

**DOI:** 10.1007/s11325-020-02263-2

**Published:** 2020-12-02

**Authors:** Anna Christina Meyer, Jens Spiesshoefer, Nina Christina Siebers, Anna Heidbreder, Christian Thiedemann, Hartmut Schneider, Andrew T. Braun, Winfried Randerath, Peter Young, Michael Dreher, Matthias Boentert

**Affiliations:** 1 Department of Neurology with Institute for Translational Neurology, Münster University Hospital, Munster, Germany; 2 Institute of Life Sciences, Scuola Superiore Sant’Anna, Pisa, Italy; 3 Department of Neurology, Medical University of Innsbruck, Innsbruck, Austria; 4 Division of Allergy, Pulmonary and Critical Care, Department of Medicine, University of Wisconsin, Madison, WI USA; 5 Sleep Disorders Center, Bayview Hospital, School of Medicine, Johns Hopkins University, Baltimore, MD USA; 6 Bethanien Hospital gGmbH Solingen, Solingen, Germany; 7 Institute for Pneumology at the University of Cologne, Solingen, Germany; 8 Medical Park Klinik Reithofpark, Bad Feilnbach, Germany; 9 Department of Pneumology and Intensive Care Medicine, University Hospital RWTH, Aachen, Germany; 10 Department of Medicine, UKM Marienhospital, Steinfurt, Germany

**Keywords:** Nasal high flow therapy, Hypercapnia, Translational respiratory physiology, Neuromuscular disorders, Sympathetic drive

## Abstract

**Purpose:**

In neuromuscular disorders (NMD), inspiratory muscle weakness may cause sleep-related hypoventilation requiring non-invasive ventilation (NIV). Alternatively, nasal high flow therapy (NHF) may ameliorate mild nocturnal hypercapnia (NH) through washout of anatomical dead space and generation of positive airway pressure. Ventilatory support by NIV or NHF might have favourable short-term effects on sympathovagal balance (SVB). This study comparatively investigated the effects of NHF and NIV on sleep-related breathing and SVB in NMD patients with evolving NH.

**Methods:**

Transcutaneous CO_2_ (p_tc_CO_2_), peripheral oxygen saturation (SpO_2_), sleep outcomes and SVB (spectral analysis of heart rate, diastolic blood pressure variability) along with haemodynamic measures (cardiac index, total peripheral resistance index) were evaluated overnight in 17 patients. Polysomnographies (PSG) were randomly split into equal parts with no treatment, NIV and NHF at different flow rates (20 l/min vs. 50 l/min). In-depth analysis of SVB and haemodynamics was performed on 10-min segments of stable N2 sleep taken from each intervention.

**Results:**

Compared with no treatment, NHF20 and NHF50 did not significantly change p_tc_CO_2_, SpO_2_ or the apnea hypopnea index (AHI). NHF50 was poorly tolerated. In contrast, NIV significantly improved both gas exchange and AHI without adversely affecting sleep. During daytime, NHF20 and NHF50 had neutral effects on ventilation and oxygenation whereas NIV improved p_tc_CO_2_ and SpO_2_. Effects of NIV and NHF on SVB and haemodynamics were neutral during both night and daytime.

**Conclusions:**

NHF does not correct sleep-disordered breathing in NMD patients with NH. Both NHF and NIV exert no immediate effects on SVB.

## Introduction

In patients with neuromuscular disorders (NMD), respiratory muscle involvement is common and a major cause of morbidity and mortality [1]. It substantially affects overall prognosis in amyotrophic lateral sclerosis (ALS) and Duchenne’s muscular dystrophy (DMD) [2, 3] but may also evolve in slowly progressive NMD, including hereditary myopathies such as late-onset Pompe disease (LOPD), myotonic dystrophy type 1 (DM1) and facioscapulohumeral dystrophy (FSHD) [4–9]. Pathophysiologically, respiratory muscle weakness leads to alveolar hypoventilation with hypercapnia which manifests during rapid eye movement (REM) sleep first [10]. With disease progression, hypoventilation and hypercapnia spread to non-REM sleep stages, eventually followed by daytime hypercapnia and type II respiratory failure [1, 10].

Non-invasive ventilation (NIV) is indicated when respiratory muscle weakness is accompanied by nocturnal hypercapnia (NH) and sleep-related symptoms [11], but long-term data on the effects of NIV in patients with slowly progressive NMD and mild NH are scarce and conflicting. For example, treatment adherence and therapeutic benefits have been reported to be low or indefinite in patients with DM1, in particular [12]. In this population, adherence to NIV has been shown to strongly depend on sleep-related symptoms and leakage of air or other respiratory-related issues [13]. With regard to other slowly progressing myopathies, no similar evidence has been collected to date, but based on own clinical observations a subset of patients with evolving NH and mild or even absent sleep-related complaints have considerable problems to tolerate nocturnal NIV. In these individuals, treatment-related inconveniences due to mask intolerance, air leakage, aerophagy and patient-ventilator dyssynchrony may be perceived as more serious than subjective symptoms of sleep-disordered breathing (SDB). As long as NH is not severe, these patients might benefit from different ventilator settings or an alternative type of ventilatory support which is—potentially—easier to tolerate. First, spontaneous BiPAP mode (with mere inspiratory support) can be used in which no respiratory rate is specified and the risk of dyssynchrony is reduced accordingly. Second, nasal high-flow (NHF) oxygen therapy could be considered as a treatment alternative since it has previously been shown to exert beneficial effects on several aspects of ventilation and gas exchange. To date, NHF oxygen therapy has mainly been used for treatment of acute hypoxic respiratory failure where it improves pulmonary gas exchange and relieves shortness of breath [14]. But also in patients with chronic obstructive pulmonary disease (COPD) and hypercapnic respiratory failure, NHF oxygen therapy has been reported to enhance oxygenation and reduce both respiratory rate and hypercapnia [14–17]. Using special nasal prongs NHF supplies warm humidified air with a high flow rate. Apart from an increase of inspiratory oxygen concentration (if O_2_ is supplemented), NHF is thought to generate flow-dependent positive airway pressure and reduce dead-space ventilation and CO_2_ rebreathing from the nasopharynx and trachea [18–21]. Only one case report has been published on the use of NHF in a patient with acute respiratory failure of neuromuscular origin [22], and no evidence is available with regard to NMD patients with mere NH or chronic hypercapnic respiratory failure. Assuming that alveolar ventilation corresponds to tidal volume minus dead-space ventilation, improvement of CO_2_ elimination can potentially be achieved by NHF in NMD patients with evolving NH.

At the same time, it can be hypothesised that alleviation of SDB beneficially impacts autonomic nervous system function or sympathovagal balance (SVB), respectively. This is based on the assumption that hypoventilation is associated with an increased sympathetic drive which is supported by a small number of experimental studies [23, 24]. Notably, an increase in respiratory rate may be related not only to diaphragm weakness and hypercapnia but also to increased sympathetic drive.

Therefore, the present study investigated the effects of NHF (without additional oxygen supplementation) on gas exchange, sleep and SVB in patients with NMD and mild to moderate NH. The effects of different flow rates (20 l/min—NHF20—vs. 50 l/min—NHF50) were compared with no treatment and NIV therapy. In addition, immediate effects of NHF20, NHF50 and NIV on SVB during the awake state at daytime were also assessed.

## Methods

### Study participants

Adult patients with NH due to genetically proven NMD were consecutively recruited from our academic sleep laboratory from April 2018 to August 2019. Inclusion criteria comprised ongoing NIV for less than 5 years, NIV use only during nocturnal sleep and no vital dependence on NIV. Diagnosis of NH and NIV had to be established in our own institution before in order to warrant reliability of clinical and baseline respiratory data. The study protocol conformed to the 1975 Declaration of Helsinki and was approved by the local authorities (Ethikkommission der Ärztekammer Westfalen Lippe und der Medizinischen Fakultät der Westfälischen Wilhelms-Universität Münster; Az. 2017-668-f-S). All subjects gave written informed consent to participate in the study. The project was registered under the German Clinical Trials Registry (drks.de Identifier: DRKS00013903 and DRKS00013905).

In all participants, diagnosis of NH had previously been established using transcutaneous capnometry (Sentec, Therwil, Switzerland), based on a peak carbon dioxide tension (p_tc_CO_2_) ≥ 50 mmHg or an increase in p_tc_CO_2_ by 10 mmHg above the awake baseline value [11, 25].

### Assessments

All subjects underwent detailed neurological assessment of motor abilities for documentation of their individual functional status. Spirometric and manometric measurements (forced vital capacity/FVC, maximum inspiratory pressure/MIP) were also included in the data set, if performed less than 6 months prior to study participation.

### Sleep studies

During the first night in the sleep laboratory, participants underwent both full polysomnography (PSG, Nihon Kohden, Rosbach, Germany) and transcutaneous capnometry. For the first half of the night (22:00 pm–02:00 am), patients did not use NIV in order to allow confirmation of NH. During the second half of the night (02:00–06:00 am), patients applied their own NIV device, and optimal ventilator settings were verified by confirming normocapnia (p_tc_CO_2_ < 50 mmHg), normoxia (SpO_2_ < 90% for less than 5 min) and an apnea hypopnea index (AHI) below 5 per hour. Validation of respiratory and sleep parameters were conducted according to standard diagnostic guidelines [26]. If necessary, ventilator settings were modified in order to achieve both normal gas exchange and AHI. During the second night, PSG was randomly divided into equal parts of about 2-h duration each with no treatment, NIV (using the previous night’s settings), NHF20 and NHF50. Prior to the second night, each subject was familiarised with both the NHF device and the non-invasive haemodynamic and SVB monitoring system.

### Non-invasive haemodynamic and SVB monitoring

SVB and haemodynamics were monitored using the Task Force Monitor® system (TFM®, CNSystems, Graz, Austria) which has previously been validated [27–31]. For assessment of SVB, diastolic blood pressure variability (dBPV) and heart rate variability (HRV) were analysed using the continuous non-invasive arterial blood pressure signal (CNAP® Technology, CNSystems, Graz, Austria; sampling rate 100 Hz) and a 3-lead electrocardiogram (sampling rate 1000 Hz), both implemented in the TFM®. Both HRV (based on continuous recording of variability in RR intervals) and dBPV were initially expressed in ms^2^ but were then normalised for total power spectra with the unit being percent. Data were computed by frequency domain analysis (adaptive autoregressive parameter AAR model) and presented as the high-frequency component (HF; 0.15–0.40 Hz), low-frequency component (LF; 0.04–0.15 Hz) and their relative ratio (LF/HF) for both HRV and dBPV. A higher LF/HF ratio indicates increased sympathetic drive as the LF component (of dBPV variability in particular) is believed to reflect sympathetic activation, and the HF component (of both HRV and dBPV) is considered to reflect parasympathetic drive [27–31].

Baroreceptor reflex sensitivity (BRS) was measured using the sequence method. The time constant of this stimulus-response relationship primarily reflects vagal but not sympathetic responsiveness (particularly with respect to the up sequences) [32].

Haemodynamic measurements included beat-to-beat systolic and diastolic blood pressure; these were continuously recorded and validated against periodic measurements obtained every 15 min by oscillometric recording from the upper contralateral arm. Transthoracic impedance measurements were used to estimate cardiac stroke volume index, cardiac index and systemic vascular resistance, from which the total peripheral resistance index was calculated (mean blood pressure divided by cardiac index). Bioimpedance-based measurements have been previously validated against invasive haemodynamic monitoring in the catheter lab [27–31].

All signals were simultaneously acquired and displayed in real time on a personal computer running DOMINO 2.9.0 software.

### In-depth analysis of SVB and haemodynamics at night

As measurements were fully attended (A.M. and J.S.), it was possible to evaluate sleep stages, respiration and both haemodynamic and SVB on-line and on a “beat to beat” basis. From each patient, four sleep segments were chosen by visual identification, each of them 10 min in duration. Each segment had to be characterised by stable N2 sleep and sinus rhythm with less than 5% ectopic beats in order in order to enable valid analysis of HRV, dBPV and BRS). Sleep segments were selected from each of the four interventions (no treatment, NIV, NHF20 and NHF50).

### Daytime measurements

After resting for 1 h in the half-supine position baseline values were obtained for p_tc_CO_2_, SpO_2_, haemodynamic and SVB measures. Thereafter, patients were exposed to NHF20, NHF50 and NIV in a randomised order. Each intervention lasted for 10 min. Interventions were separated by periods of spontaneous breathing of 3-min minimum until heart rate, blood pressure, p_tc_CO_2_ and SpO_2_ had returned to baseline. At baseline and following each intervention, the patients were asked to judge their subjective wellbeing on a numerical analogue scale ranging from 0 to 10, with “0” reflecting the worst and “10” indicating the best possible subjective wellbeing.

### Statistical analysis

All analyses were performed using IBM SPSS® software v.25 (IBM Deutschland GmbH, Ehningen, Germany). Assuming a two-sided significance level of 0.05 (alpha) and 80% power (beta), a sample size of 15 patients in total was calculated for detection of a 2-mmHg change in p_tc_CO_2_ (which was considered to have potential clinical relevance). Mean values and standard deviations of p_tc_CO_2_ were derived from own preliminary data. Results were expressed as mean and standard deviation for continuous variables with a rather normal distribution, and median and interquartile range for continuous variables with a rather skewed distribution. Categorical variables were expressed as percentages, unless otherwise specified. Respiratory parameters, SVB and haemodynamic parameters derived during the different interventions were compared with baseline parameters (“no treatment” intervention) using a paired *t* test or Wilcoxon rank-sum test, as appropriate. Comparisons between values derived from all four interventions were performed using the Kruskal-Wallis test for non-parametric data or univariate analysis of variance (ANOVA) for normally distributed values. For all tests, a *p* value ≤ 0.05 was considered statistically significant. For significant *p* values, Bonferroni’s post hoc correction for multiple comparisons was applied.

## Results

A total of 17 patients were enrolled in the study. Table 1 summarises demographic and clinical characteristics of the study population. Neuromuscular disease entities comprised DM1 (*n* = 7), LOPD (*n* = 2), FSHD (*n* = 2), limb-girdle muscular dystrophy (*n* = 1), congenital myasthenic syndrome (*n* = 1), mitochondrial myopathy (*n* = 1), amyotrophic lateral sclerosis with slow disease progression (*n* = 1) and spinal and bulbar muscular atrophy (*n* = 1). One patient (male, 35 years of age) carried pathogenic mutations for both FSHD and DM1. Following daytime testing 16 out of 17 NMD patients participated in nocturnal assessment of NHF and NIV effects. Consistent with the previous diagnosis of NH, mean and peak p_tc_CO_2_ were increased without NIV during night 1 (Table 1). During the same night and without NIV, 4 (25.0%) patients showed moderate or severe obstructive sleep apnea as reflected by an AHI > 15/h. Using NIV, 6 out of 16 patients (37.5%) showed normocapnia and normalization of the AHI throughout the intervention. In the remaining subjects, either pressure settings or minute volume had to be titrated in order to correct mild residual NH or sleep apnea, respectively.

**Table 1:** Demographic and baseline characteristics of the study population

	NMD patients (*n* = 17)
Male, *n* (%)	10 (58.8)
Age, years	47.3 ± 14.4
BMI, kg/m^2^	24.8 ± 6.1
BSA, m^2^	1.9 ± 0.3
FVC (% of normal value)	61.6 ± 24.4
MEP (% of reference value)	47.6 ± 15.2
MIP (% of reference value)	47.7 ± 23.0
Peak cough flow (l/min)	246.7 ± 101.0
AHI/h	10.6 ± 8.3
*t* < 90% (min)	12.7 (3.9–70.2)
Min SpO_2_	80.4 ± 10.5
Mean SpO_2_	91.4 ± 4.5
Mean p_tc_CO_2_	47.5 ± 3.7
Peak p_tc_CO_2_	53.4 ± 4.0

Values are expressed as mean and standard deviation, median (interquartile range) or number of patients (%)

*BMI* body mass index, *BSA* body surface area, *FVC* forced vital capacity, *MEP* maximal expiratory pressure, *MIP* maximal inspiratory pressure, *AHI* apnoea hypopnoea index, *SpO*_*2*_ peripheral oxygen saturation, *p*_*tc*_*CO*_*2*_ transcutaneous carbon dioxide

### Impact of NHF20 on sleep, ventilation and sympathovagal balance (night 2)

Objective sleep outcomes are depicted in Table 2 showing no statistical differences between the various interventions. Compared with no treatment, NHF20 did not lead to significant improvement of p_tc_CO_2_ and AHI (Table 3). Effects of NHF20 on SVB and haemodynamic measures during N2 sleep were neutral compared with no treatment (Table 4).

**Table 2:** Effects of NHF and NIV on sleep outcomes in NMD patients at night

Duration of intervention, min [%TIB]	No treatment (*n* = 16)	NHF 20 (*n* = 16)	*p*^1)^	NHF 50 (*n* = 11)	p^1)^	NIV (*n* = 16)	*p*^1)^	*p*^2)^
	115.5 (91.0–126.0) [24.4 (20.0–29.0)]	124.0 (111.5–138.5) [27.6 (26.7–30.0)]	n.s.	88.5 (60.0–102.5) [18.6 (0.0–21.3)]	n.s.	136.0 (112.5–199.5) [28.1 (24.3–40.6)]	n.s.	0.001^§^
Sleep outcomes								
TST (% of intervention period)	74.2 (53.7–84.4)	63.7 (42.7–80.9)	n.s.	45.6 (1.7–69.4)	0.004	72.9 (57.9–85.0)	n.s.	0.001^§^
N1 (% of intervention period)	4.6 (2.6–7.2)	3.9 (1.6–10.1)	-	3.0 (1.5–5.4)	-	2.3 (1.7–3.1)	-	n.s.^§^
N2 (% of intervention period)	23.6 (17.7–32.3)	33.2 (20.1–36.1)	-	26.3 (0.0–34.1)	-	32.9 (24.5–48.5)	-	n.s.
N3 (% of intervention period)	26.8 (24–34.5)	9.9 (0.0–25.4)	0.019	2.9 (0.0–25.0)	0.014	17.5 (10.7–28.9)	n.s.	0.023^§^
REM (% of intervention period)	2.6 (0.0–17.2)	9.7 (0.6–14.1)	n.s.	0.0 (0.0–0.0)	n.s.	13.1 (7.3–15.8)	n.s.	0.001^§^
Sleep efficiency, % TST/TIB	63.7 (55.9–91.0)	60.7 (39.7–84.7)	-	55.9 (11.5–76.1)	-	74.5 (53.0–92.5)	-	n.s.^§^
Arousal index, /h	14.5 (8.7–21.3)	18.6 (13.2–36.5)	-	15.2 (9.9–56.1)	-	10.6 (8.0–16.6)	-	n.s.^§^
Resp arousals, /h	1.8 (0.0–6.3)	2.5 (1.4–17.1)	-	0.0 (0.0–19.4)	-	0.6 (0.0–2.1)	-	n.s.^§^

Values are expressed as mean and standard deviation or median and interquartile range (in brackets). Post hoc testing was not performed when results of multigroup comparisons were non-significant

*NHF* nasal high flow, *NIV* non-invasive ventilation, *TIB* time in bed, *TST* total sleep time, *N3* slow wave sleep, *REM* rapid eye movement sleep

^1)^*p* values for comparison with baseline values (“no treatment”) using the Wilcoxon rank-sum test

^2)^*p* values for comparison between all groups/variables

^§^Kruskal-Wallis test

**Table 3:** Effects of NHF and NIV on respiratory outcomes in NMD patients at night

	No treatment (*n* = 16)	NHF 20 (*n* = 16)	*p*^1)^	NHF 50 (*n* = 11)	*p*^1)^	NIV (*n* = 16)	*p*^1)^	*p*^2)^
AHI, /h	9.5 (0.7–16.5)	6.8 (2.5–31.2)	-	20.7 (1.1–28.9)	-	2.3 (0.0–3.9)	-	n.s.^§^
AI, /h	1.2 (0.0–13.8)	4.0 (0.4–21.3)	-	19.3 (1.1–28.9)	-	0.2 (0.0–3.1)	-	n.s.^§^
cAI, /h	0.0 (0.0–0.8)	0.3 (0.0–6.9)	-	1.2 (0.0–13.9)	-	0.0 (0.0–0.9)	-	n.s.^§^
oAI, /h	0.0 (0.0–5.4)	1.0 (0.0–8.0)	-	0.9 (0.0–7.3)	-	0.0 (0.0–0.0)	-	n.s.^§^
HI, /h	0.9 (0.0–6.5)	2.2 (0.4–5.4)	-	0.0 (0.0–0.6)	-	0.2 (0.0–1.5)	-	n.s.^§^
ODI, /h	5.5 (0.7–13.0)	2.7 (0.8–21.2)	-	0.9 (0.0–25.4)	-	1.0 (0.0–1.6)	-	n.s.^§^
Mean SpO_2_ (%)	94.1 ± 2.8	94.1 ± 4.0	-	94.3 ± 3.9	-	95.6 ± 2.5	-	n.s.^&^
Minimum SpO_2_, %	86.2 ± 6.9	85.9 ± 11.2	-	86.1 ± 11.4	-	84.6 ± 11.2	-	n.s.^&^
*t* < 90%, min	1.6 (0.0–17.0)	1.1 (0.0–5.9)	-	0.4 (0.0–17.2)	-	0.3 (0.0–3.5)	-	n.s.^§^
Mean p_tc_CO_2_, mmHg	45.5 ± 4.7	44.9 ± 4.2	n.s.	43.1 ± 6.5	n.s.	39.9 ± 3.5	0.007	0.063^&^
Peak p_tc_CO_2_, mmHg	50.1 ± 6.6	50.1 ± 7.9	n.s	46.4 ± 6.6	n.s.	44.8 ± 2.6	0.001	0.009^&^

Values are based on the actual duration of each intervention (no treatment, NHF20, NHF50, NIV). Values are expressed as mean and standard deviation or median and interquartile range (in brackets). Post hoc tests were not performed when results of multigroup comparisons were non-significant. As an exception, the *p* value is reported for the post hoc *t* test comparing mean p_tc_CO_2_ between the NIV and “no treatment” period

*NHF* nasal high flow, *NIV* non-invasive ventilation, *AHI* apnoea-hypopnea index, *AI* apnoea index, *cAI* central apnea index, *oAI* obstructive apnoea index, *HI* hypopnea index, *ODI* oxygen desaturation index, *SpO*_*2*_ peripheral oxygen saturation, *t < 90%* duration of oxygen desaturation ≥ 3%, *p*_*tc*_*CO*_*2*_ transcutaneous carbon dioxide tension

^1)^*p* values for comparison with baseline values (“no treatment”) using either the paired *t* test or the Wilcoxon rank-sum test as appropriate

^2)^*p* value for comparison between all groups/variables

^§^Kruskal-Wallis test

^&^Univariate ANOVA

**Table 4:** Effect of NHF and NIV on sympathovagal balance and haemodynamics during stable N2 sleep in NMD patients

	No treatment (*n* = 16)	NHF 20 (*n* = 16)	*p*^1)^	NHF 50 (*n* = 7)	*p*^1)^	NIV (*n* = 14)	*p*^1)^	*p*^2)^
Sympathovagal balance								
HFnuRRI, %	44.8 ± 20.4	48.3 ± 21.7	–	43.7 ± 18.9	–	44.2 ± 22.3	–	n.s.^&^
LFnuRRI, %	55.2 ± 20.4	51.7 ± 21.7	–	56.3 ± 18.9	–	55.8 ± 22.3	–	n.s.^&^
LF/HF RRI	1.1(0.9–3.2)	1.3 (0.7–2.6)	–	1.3 (1.2–2.1)	–	1.6 (1.0–2.5)	–	n.s.^&^
HFnudBPV, %	15.3 ± 6.8	13.5 ± 6.7	–	14.3 ± 6.4	–	11.2 ± 7.5	–	n.s.^&^
LFnudBPV, %	45.3 ± 10.0	46.3 ± 11.7	–	40.9 ± 15.8	–	43.5 ± 12.1	–	n.s.^&^
LF/HF dBPV	3.4 (1.7–5.0)	4.0 (2.2–5.8)	–	2.5 (1.8–4.2)	–	4.4 (2.7–7.3)	–	n.s.^&^
BRS slope								
Up event counts	11.5 ± 8.5	13.1 ± 7.6	–	17.3 ± 8.3	–	11.5 ± 7.6	–	n.s.^&^
Up events, ms/mmHg	14.8 ± 8.4	20.7 ± 14.7	–	27.0 ± 14.5	–	14.8 ± 14.1	–	n.s.^&^
Down event counts	17.9 ± 12.9	20.3 ± 13.4	–	26.0 ± 9.8	–	11.6 ± 7.9	–	n.s.^&^
Down events, ms/mmHg	15.5 ± 10.81	23.5 ± 22.4 (10.3–24.8)	–	29.4 ± 18.5	–	16.4 ± 24.2	–	n.s.^&^
Haemodynamic parameters								
Heart rate, min^−1^	63.6 ± 10.5	62.9 ± 8.5	–	57.9 ± 17.1	–	61.2 ± 7.5	–	n.s.^&^
Systolic BP, mmHg	102.3 ± 19.7	102.3 ± 17.2	–	97.2 ± 21.2	–	100.7 ± 21.4	–	n.s.^&^
Diastolic BP, mmHg	65.9 ± 16.4	65.5 ± 14.0	–	60.9 ± 12.5	–	61.9 ± 17.5	–	n.s.^&^
Stroke volume index, ml/beat/m^2^ L/m^2^	33.5 ± 10.0	32.5 ± 8.7	–	32.6 ± 8.5	–	33.4 ± 10.1	–	n.s.^§^
Cardiac index, L/min/ m^2^	2.1 ± 0.6	2.1 ± 0.6	–	1.9 ± 0.3	–	2.0 ± 0.4	–	n.s.^§^
TPRI, dyne·s m^2^ cm^−5^	3253.9 ± 1259.0	3363.2 ± 1165.0	–	3155.4.8 ± 862.6	–	3274.7 ± 1243.2	**–**	n.s.^§^

Values are expressed as mean and standard deviation or median and interquartile range (in brackets) for sleep segments of 10-min duration taken from stable N2 sleep with sinus rhythm. Post hoc tests were not performed when results of multigroup comparisons were non-significant. Analysis of BRS data is based on 9/12 patients for up events and 10/12 patients for down events, respectively

*NHF* nasal high flow, *NIV* non-invasive ventilation, *BRS* baroreceptor reflex sensitivity (up events and down events), *HFnudBPV* high-frequency component of diastolic blood pressure variability, *HFnuRRI* high-frequency component of heart rate variability, *LFnudBPV* low-frequency component of diastolic blood pressure variability, *LF/HF dBPV* relative ratio of low frequency and high frequency component of diastolic blood pressure variability, *LFnuRRI* low-frequency component of heart rate variability, *LF/HF RRI* relative ratio of low-frequency and high-frequency component of heart rate variability, *n.s.* not statistically significant, *TPRI* total peripheral resistance index

^1)^*p* values for comparison with baseline values (“no treatment”) using either the paired *t* test or the Wilcoxon rank-sum test as appropriate

^2)^*p* value for comparison between all groups/variables

^§^Kruskal-Wallis test

^&^Univariate ANOVA

### Impact of NHF50 on sleep, ventilation and sympathovagal balance (night 2)

In a substantial number of participants, NHF50 was poorly tolerated with 5 out 16 NMD patients eventually declining treatment and 4 patients not showing consecutive 10 min of stable N2 sleep (Table 2). Mean duration of NHF50 usage was significantly lower than with NHF20 or NIV, and compared with no treatment, percentage of N3 and REM sleep was reduced (Table 2). During the NHF50 intervention peak p_tc_CO_2_ was lower than with no treatment in the few patients tolerating the intervention (Table 3). Effects on SVB during N2 sleep were neutral compared with the no-treatment period (Table 4). Haemodynamic effects were also neutral (Table 4).

### Impact of NIV on sleep, ventilation and sympathovagal balance (night 2)

Non-invasive ventilation improved nocturnal hypercapnia as reflected by a significant decrease of mean and maximum p_tc_CO_2_ when compared with the “no treatment” period (Table 3; Cohen’s d 1.35 and 1.06, respectively).. Compared with “no treatment”, the percentage of REM sleep was higher during the NHF20 and NIV interventions but post hoc tests were not significant (Table 2). Since a subset of patients early requested cessation of NHF50 and continued with NIV, overall duration of NIV usage was longer than with NHF interventions (Table 2). Different from both NHF interventions, NIV improved mean and maximum p_tc_CO_2_ (Table 3). Effects of NIV on haemodynamics and sympathovagal balance were all neutral (Table 4).

### Immediate effects of NHF20, NHF50 and NIV on respiration and sympathovagal balance at daytime

In the awake state during daytime, NHF20 and NHF50 led to a significant and flow-dependent decrease in subjective well-being (Table 5). At the same time, the effect of NHF20 and NHF50 on p_tc_CO_2_ was neutral. Again NIV showed favourable effects on respiration as reflected by a significant decrease of p_tc_CO_2_ (Table 5). Haemodynamic effects were neutral (Table 5).

**Table 5:** Effect of NHF20 and NHF50 on sympatho-vagal balance and haemodynamic parameters in awake NMD patients during daytime

	Baseline	NHF 20	*p*^1)^	NHF 50	*p*^1)^	NIV	*p*^1)^	*p*^2)^
Well-being score (10–0)	8.1 ± 1.7	6.2 ± 2.4	0.01	4.6 ± 3.0	<0.01	7.6 ± 1.6	0.33	0.01^&^
Respiratory parameters								
Mean SpO_2_, %	95.0 ± 2.4	94.4 ± 2.5	–	94.4 ± 3.4	–	96.3 ± 1.4	–	n.s.^&^
Mean p_tc_CO_2_, mmHg	42.1 ± 4.4	41.9 ± 4.8	n.s.	42.5 ± 5.4	n.s.	37.7 ± 3.4	0.013	0.026^&^
Sympatho-vagal balance parameters								
HFnuRRI, %	44.9 ± 12.6	46.2 ± 15.8	–	45.9 ± 17.8	–	44.2 ± 15.7	–	n.s.^&^
LFnuRRI, %	55.1 ± 12.6	53.8 ± 15.8	–	54.1 ± 17.8	–	55.8 ± 15.7	–	n.s.^&^
LF/HF RRI	1.6 ± 0.9	1.8 ± 1.5	–	1.9 ± 1.6	–	1.8 ± 1.1	–	n.s.^&^
HFnu dBPV, %	20.0 ± 12.3	18.1 ± 7.6	–	16.8 ± 6.4	–	19.8 ± 8.2	–	n.s.^&^
LF nudBPV, %	41.4 ± 12.8	42.2 ± 10.7	–	43.1 ± 10.1	–	41.8 ± 11.0	–	n.s.^&^
LF/HF dBPV	3.3 ± 3.0	3.2 ± 2.9	–	3.4 ± 2.6	–	2.6 ± 1.5	–	n.s.^&^
BRS slope								
Up event count, n ^ Up event count, n ^	13.6 ± 10.9 13.6 ± 10.9	15.1 ± 10.3 15.1 ± 10.3	–	17.0 ± 12.1 17.0 ± 12.1	–	10.2 ± 8.5 10.2 ± 8.5	–	n.s.^&^
Up events, ms/mmHg	13.8 ± 8.1	19.8 ± 13.1	–	19.4 ± 17.3	–	17.0 ± 18.6	–	n.s.^&^
Down event count, *n* ^	14.4 ± 10.5	18.8 ± 14.6	–	17.9 ± 9.7	–	13.3 ± 10.8	–	n.s.^&^
Down events, ms/mmHg	15.1 ± 7.6	17.6 ± 10.2	–	18.9 ± 14.9	–	17.6 ± 13.4	–	n.s.^&^
Haemodynamic parameters								
Heart rate, min^−1^	70.2 ± 9.0	66.4 ± 8.2	**–**	65.7 ± 9.6	**–**	65.8 ± 8.8	**–**	n.s.^&^
Systolic BP, mmHg	115.4 ± 12.8	110.4 ± 12.4	–	111.5 ± 18.9	–	107.7 ± 17.7	–	n.s.^&^
Diastolic BP, mmHg	73.1 ± 9.6	71.5 ± 10.4	–	72.8 ± 14.1	–	69.9 ± 15.6	–	n.s.^&^
Stroke volume index, mL/m^2^	37.5 ± 10.8	37.6 ± 9.5	–	37.0 ± 9.3	–	36.9 ± 9.0	–	n.s.^&^
Cardiac index, L/min/ m^2^	2.5 ± 0.7	2.5 ± 0.6	–	2.4 ± 0.5	–	2.4 ± 0.5	–	n.s.^&^
TPRI, dyne·s m^2^ cm^−5^	3006.4 ± 978.8	2932.8 ± 929.1	–	3027.5 ± 886.6	–	2918.3 ± 907.7	–	n.s.^&^

Values are depicted as mean and standard deviation or median (interquartile range) for sleep segment of 10-min duration taken from stable N2 sleep with sinus rhythm. Post hoc *t* tests were not performed when results of multigroup comparisons were non-significant

*NHF* nasal high flow, *NIV* non-invasive ventilation, *BRS slope* slope of baroreceptor reflex sensitivity (up events and down events), *HFnudBPV* high-frequency component of diastolic blood pressure variability, *HFnuRRI* high-frequency component of heart rate variability, *LFnudBPV* low-frequency component of diastolic blood pressure variability, *LF/HF dBPV* relative ratio of low-frequency and high-frequency component of diastolic blood pressure variability, *LFnuRRI* low-frequency component of heart rate variability, *LF/HF RRI* relative ratio of low-frequency and high-frequency component of heart rate variability, *nu* normalised units (normalised for total power spectra), *n.s.* not statistically significant, *TPRI* total peripheral resistance index

^1)^*p* values for comparison with baseline values (“no treatment”) using the paired test

^2)^*p* values for comparison between all groups/variables

^&^Univariate ANOVA

## Discussion

This is the first study which explores the effects of NHF on SDB and sleep in adult patients with slowly progressing NMD and isolated NH. Specifically, it evaluates whether NHF improves CO_2_ elimination and upper airway patency depending on the flow rate applied. Finally, this study assessed tolerability of nocturnal NHF and its effects on SVB during N2 sleep as compared with NIV.

From a practical point of view, this study aimed to evaluate whether or not NHF can be considered a therapeutic option for NMD patients with evolving NH who do not tolerate NIV or who use NIV during nocturnal sleep but seek for a different kind of ventilatory support for intermittent daytime use.

The main findings of this study can be summarised as follows:

(i) Independent on the flow rate applied, NHF does not improve SDB in patients with NMD. Both alveolar hypoventilation during sleep and obstructive sleep apnea (if present) are not sufficiently treated by application of NHF. In contrast, NIV proved to successfully normalise NH and sleep apnea.
(ii) Nasal high flow therapy at 20 l/min, and at 50 l/min in particular, is poorly tolerated and disrupts sleep whereas NIV proves to enhance objective sleep quality along with effective treatment of SDB.
(iii) Effects of NHF and NIV on autonomous nervous system activity are mainly neutral during sleep and in the awake state. Both interventions do not cause a measurable increase of sympathetic drive.

### Impact of NHF on sleep-related breathing

The fact that NHF does not exert beneficial effects on sleep-related breathing in patients with NMD suggests that mechanistic explanations for the improvement of gas exchange and upper airway stability by NHF cannot be proven in this subgroup. First, NHF apparently does not build up sufficient positive airway pressure to sufficiently stabilise the upper airway and to substantially reduce obstructive events. This finding is consistent with previous studies that showed that every 10 l of increase in NHF flow rate translate into a ~ 1 cmH_2_O increase in positive airway pressure [19]. Second, the assumed reduction of dead-space ventilation due to NHF did not lead to a measurable improvement of CO_2_ elimination. Previous studies have shown that NHF slows down respiratory rate and reduces dead-space ventilation, tidal volume and minute ventilation in healthy subjects during sleep [33]. With regard to patients, reduction of respiratory rate appears to be the most ostensible effect of NHF in adults and children [34, 35]. However, data on the effect of NHF on CO_2_ tension are still conflicting. Continuous use of NHF (30 l/min flow rate) for 8 h has been shown to significantly lower capillary pCO_2_ in awake patients with COPD by 0.69 ± 0.2 kPa or 5.18 ± 1.5 mmHg, respectively [34]. In contrast, transcutaneously measured tissue CO_2_ did not fall after 10 min of NHF20 despite significant reduction of dead space [21]. Taking into account that NHF builds up only little positive airway pressure (~ 2 cmH_2_O with NHF20 and ~ 5 cmH_2_O with NHF50)—that, in addition, remains stable throughout the respiratory cycle—it seems plausible that NHF could not correct NH in this study. It should be acknowledged that NHF50 appeared to improve exhalation of CO_2_ in those few NMD patients who did tolerate the intervention. However, differences did not reach significance most likely due to the small sample size.

In summary, the mechanistic effects of NHF appear to be insufficient to improve upper airway patency and CO_2_ exhalation in patients with NMD. In this context, it has to be considered as a major methodological aspect that the present study did not apply nasal high flow *oxygen* therapy but NHF alone. Combination of NHF with O_2_ supplementation may predominantly account for improved oxygenation and reduction of respiratory rate which both have been reported in adult patients with acute respiratory failure and COPD. In children, low positive airway pressure levels as achievable by NHF may be sufficient to meaningfully reduce obstructive events [36–40].

### Tolerability of NHF50

Application of NHF50 was poorly tolerated by patients with NMD in the present study. Tolerability was low both in the awake state during daytime and during nocturnal sleep. This finding seems to contradict previous studies that showed improvement of subjective well-being by NHF oxygen therapy with higher flow rates in patients with COPD, NMD or heart failure [14, 16, 18, 22, 41]. Two aspects may help explain these discrepant findings. First, beneficial effects of NHF were previously made in patients with acute dyspnea or in patients declining NIV therapy. In contrast, tolerability of NHF50 may obviously be hampered in non-dyspneic patients who mainly experience significant discomfort due to noise and nasal flow sensation. Second, improved oxygenation is likely perceived as directly relieving by patients with COPD or heart failure in whom hypoxic respiratory failure is also present. In particular, correction of hypoxia lowers sympathetic drive and reduces respiratory rate both leading to more efficient ventilation and clearance of CO_2_.

### Impact of NHF or NIV on sympathovagal balance

In this study, it could not be confirmed that application of either NHF or NIV translates into a decrease in sympathetic drive. Neutral effects of NHF on autonomous nervous system activity may simply be explained by the fact that SDB was not significantly alleviated. Furthermore, study participants showed sleep disruption—especially with NHF 50—which may have negated any positive effects on SVB.

With regard to the effects of nocturnal NIV on autonomous nervous system activity this study could not show any robust improvement although both hypoventilation and hypercapnia during sleep were adequately treated. However, study participants were already used to NIV before which might have been associated with better tolerability of this intervention in this study.

The results of this study suggest that patients with NMD and mild to moderate NH do not represent appropriate candidates for NHF. First, these patients usually do not present with hypoxia and acute or chronic dyspnea which are linked to increased sympathetic nerve activity. Second, these patients do not show a rapid shallow breathing pattern whose correction by NHF translates into reduced work of breathing and hence sympathetic drive.

### Study limitations

The present study has several limitations that have to be taken into account. Firstly, the number of patients enrolled was too small to statistically deduce evidence which can reliably be generalised. However, this study aimed to provide (or falsify) a first-ever proof of principle regarding the use of NHF in NMD patients with NH. Secondly, duration of each intervention was a priori limited to about 2 h. It cannot be excluded that longer periods of NHF would have exerted different effects on the variables evaluated in this study. Thirdly, sympathetic drive was not measured invasively, e.g. by direct recording of muscle sympathetic nerve activity. Non-invasive recording of HRV and dBPV can only provide an overall estimate of SVB, including also the vagal component. However, close correlation between muscle sympathetic nerve activity and HRV/dBPV measures (LF/HF ratios in particular) has previously been shown [42, 43].

Finally, tolerability of NHF was potentially hampered by the fact that patients were confronted with this kind of ventilatory support for the first time. At the same time, low tolerability of NHF50 in particular is in line with one recent previous report on this in adult patients with obstructive sleep apnoea [44]. In contrast, all patients had started NIV substantially earlier including the opportunity to get used to it. In order to account for this assumption, we thoroughly introduced NHF to study participants prior to the daytime recordings. However, study results may be limited with regard to sleep quality measures and SVB parameters, in particular.

## Conclusion

The present study speaks out against the use of NHF as a treatment alternative to NIV in non-dyspneic NMD patients with mild to moderate NH or obstructive sleep apnea. NHF appears to be less tolerable than bi-level ventilatory support for patients with previously established NIV. Conventional NIV sufficiently corrects sleep-related hypercapnia in these patients without adverse effects on sleep or sympathetic drive.
